# Neglected Tropical Diseases Remain a Considerable Public Health Challenge in West Africa

**DOI:** 10.3390/tropicalmed10030077

**Published:** 2025-03-14

**Authors:** Andrew Ramsay, Edward Mberu Kamau

**Affiliations:** 1 Division of Infection and Global Health, St. Andrews University Medical School, St. Andrews KY16 9TF, UK; 2 UNICEF/UNDP/World Bank/WHO Special Programme for Research and Training in Tropical Diseases (TDR), World Health Organization, 1211 Geneva, Switzerland; kamaued@who.int

## 1. Neglected Tropical Diseases and the WHO NTD Roadmap

Neglected tropical diseases (NTDs) form a category of diverse, mainly infectious, diseases that are prevalent in tropical and subtropical countries. They cause significant ill health, economic hardship, disability and death and are associated with stigma and discrimination. Impoverished communities in rural areas with poor access to healthcare, clean water and sanitation are disproportionately affected, with growing numbers of people being put at risk due to population displacement and climate change [1]. These diseases, furthermore, effect major macro-economic impacts, costing low- and middle-income countries billions of USD each year and driving vicious cycles of poverty [2]. NTDs currently comprise 21 different diseases or groups of related diseases (see Table 1, Table 2 and Table 3).

They are considered ‘neglected’ tropical diseases because they have hitherto not received the attention required to combat them as public health issues. This is due to a lack of funding and interest, in no small part, because the diseases predominantly affect poor communities in marginalized areas. Historically (and, in some instances, currently), NTD patients’ poverty, coupled with their communities’ and countries’ lack of wealth, has served as a disincentive to the development of effective drugs, diagnostics and vaccines in the biotechnology and pharmaceutical industries. However, recent international public health coalitions and public–private product development partnerships, particularly those promoted by the World Health Organization (WHO), have perceptibly changed this landscape. In some cases, generous donations of drugs for NTD control programs from pharmaceutical companies have had crucial impacts.

Since all NTDs are potentially treatable and/or preventable, they can be controlled (defined as a reduction in disease incidence, prevalence, morbidity and/or mortality to a locally acceptable level as a result of deliberate efforts) [31]. In 2020, the WHO published a 10-year plan for controlling NTDs, titled ‘Ending the neglect to attain the Sustainable Development Goals; a roadmap for neglected tropical diseases 2021–2030’ [32]. As well as establishing a strategy for managing NTDs, the document lays out more ambitious goals for 13 NTDs, including the following:

(a) Eradication—a permanent reduction in the worldwide incidence of a specific pathogen to zero, due to deliberate efforts, with no risk of reintroduction;
(b) Elimination (interruption of transmission)—a reduction in the incidence of infection caused by a specific pathogen in a defined geographical area to zero, with a minimal risk of reintroduction, due to deliberate efforts;
(c) Elimination as a public health problem—the achievement of measurable targets set by the WHO in relation to a specific infection/disease [31].

Two NTDs are targeted for eradication by 2030, three for elimination (interruption of transmission), eight for elimination as public health problems and nine for control. The total numbers for eradication, elimination and control do not add up to 21 as two diseases, Human African Trypanosomiasis (HAT) and leishmaniasis have different targets for different forms of the disease. Due to ecological differences, East African HAT (caused by *Trypanosoma brucei rhodesiense*) is intended to be eliminated as a public health problem while West African HAT (caused by *T.b. gambiense*) is targeted for elimination (interruption of transmission). Similarly, visceral leishmaniasis is to be eliminated as a public health problem while cutaneous leishmaniasis is to be controlled. Noma (also known as cancrum oris) was only recently included in the WHO’s list of NTDs (December 2023), and efforts are ongoing to integrate it into the NTD roadmap. Thus, no goal has yet been set for noma. Further details on the goals for each disease are provided in Table 1, Table 2 and Table 3. The WHO recognizes that control and elimination will require sustained action beyond the attainment of the goals to maintain them.

This comprehensive 2021–2030 NTD roadmap is a product of extensive consultation and thus reflects the perspectives of the WHO Member States and a broad range of stakeholders, including those engaged in NTD prevention and control in affected countries, disease experts from various disciplines, donors and partners. Built around three ‘pillars’ (Box 1), it details the strategies and integrated approaches required to attain the defined goals in relation to the control, elimination or eradication of NTDs. Furthermore, it delineates milestones and indicators for measuring progress towards these goals. It specifies not only overarching and disease-specific but also cross-cutting indicators and targets that are aligned with the WHO’s 13th General Program of Work (2019–2023) and the Sustainable Development Goals (SDGs).

Box 1 **The NTD Roadmap Pillars.**

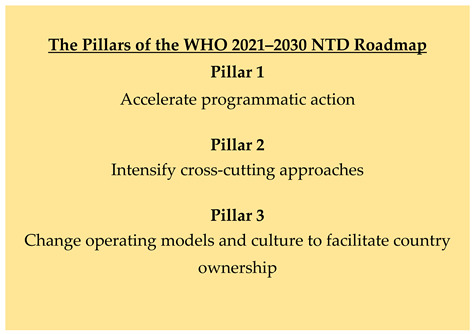

## 2. NTDs and West Africa

With the exception of chagas disease (‘South American Trypanosomiasis’), all NTDs are of major public health importance in Africa and nowhere more so than West Africa, where diseases such as onchocerciasis (or river blindness) have caused significant morbidity, disability and death.

West Africa (or Western Africa) is a subregion defined by the United Nations (UN) as the 16 countries of Benin, Burkina Faso, Cape Verde, Cote d’Ivoire, The Gambia, Ghana, Guinea, Guinea-Bissau, Liberia, Mali, Mauritania, Niger, Nigeria, Senegal, Sierra Leone and Togo. The Western African region of the African Union (AU), on the other hand, comprises only 15 countries, which are the same as those listed by the UN but exclude Mauritania, which is in the AU Northern Africa region. For the purposes of this manuscript, we consider the more inclusive UN definition. Of the sixteen countries, eight are classified as low-income countries by the World Bank (Burkina Faso, Gambia, Guinea, Guinea-Bissau, Liberia, Mali, Sierra Leone and Togo). The remaining eight countries (Benin, Cabo Verde, Cote d’Ivoire, Ghana, Mauritania, Niger, Nigeria and Senegal) are classified as low–middle-income countries.

Table 1, Table 2 and Table 3 present the 21 NTDs, the WHO roadmap goals and the current status of each disease in the 16 countries of West Africa.

It should be emphasised that there are also disease-specific, or disease group-specific, roadmaps and plans for specific NTDs or groups of NTDs. These include, for example, the strategic framework for the integrated control and management of ‘skin NTDs’, those 10 or more that induce changes in the skin before predominantly progressing to damage internal organs or cause physical disability. The 2022 WHO document ‘Ending the Neglect to Attain the Sustainable Development Goals: a strategic framework for integration and management of skin-related neglected tropical diseases’ was produced as a companion document to the broader NTD roadmap.

## 3. The Progress of West African Countries Toward the Goals, Indicators and Targets of the WHO Roadmap

As of 2025, we stand almost midway in the 2021–2030 roadmap’s timeframe. There have been major achievements, both globally and in West Africa, in controlling or eliminating the public health impact of NTDs. Globally, both yaws and dracunculiasis are close to eradication. Among the 16 countries of West Africa, one country has eliminated four NTDs either as public health problems or by interrupting disease transmission, two have eliminated three NTDs, one has eliminated two NTDs and seven have eliminated one NTD (see Table 1, Table 2, Table 3 and Table 4). However, it is acknowledged, in both the global and regional/sub-regional contexts, that there have been significant challenges, and some of these are ongoing. West Africa is not exempt from these; five of the sixteen West African countries have yet to eliminate a single NTD. Moreover, while advancement toward many of the goals is on track or has achieved (or even surpassed) the WHO’s 2030 targets, there are many areas where countries are either not collecting or not presenting data to document their development toward the agreed indicators. There are also several indicators, in all countries, where progress has not reached the half-way points toward the 2030 targets that might be expected at this stage in the roadmap’s timeframe. Table 4 outlines the considerable advancements made by the 16 countries toward the indicators and targets and highlights (in red) areas where data are not available or where the half-way point has not been reached.

## 4. Meeting the Challenges in NTD Control and Elimination Through Operational Research

Both the NTD roadmap and companion documents such as the strategic framework for skin-related NTDs point to the significance of operational research in tackling programmatic constraints and critical gaps in knowledge. Both documents emphasize the need for operational research in countries that are endemic for NTDs and advocate the strengthening of local capacity to realize this. Both documents also catalog suggested operational research priorities. The establishment of the Coalition for Operational Research on NTDs (COR-NTD) as a leading scientific body with an NTD focus has been hailed as exemplifying how NTD structures can be strengthened and cross-sectoral collaboration can be fostered. COR-NTD is among a number of bodies that provide funding for operational NTD research.

Currently, the WHO is undertaking a broad-based, consensus-building initiative among NTD stakeholders to develop a blueprint for research and development in the field of NTDs [33]. The aim is to produce a ranked list of research priorities (including operational and implementation research priorities) that will allow NTD programs, researchers and their funders to dedicate available time and financial resources to the topics of greatest urgency. This document, ‘R&D for the Overlooked: Restoring hope one research question at a time’, will be published in 2025. This blueprint will be of great value, but national NTD programs must also determine their own research priorities; they need not wait for a global blueprint if crucial research questions relevant to policy and/or practice are identified through experiences in their own work.

**Figure 1:**
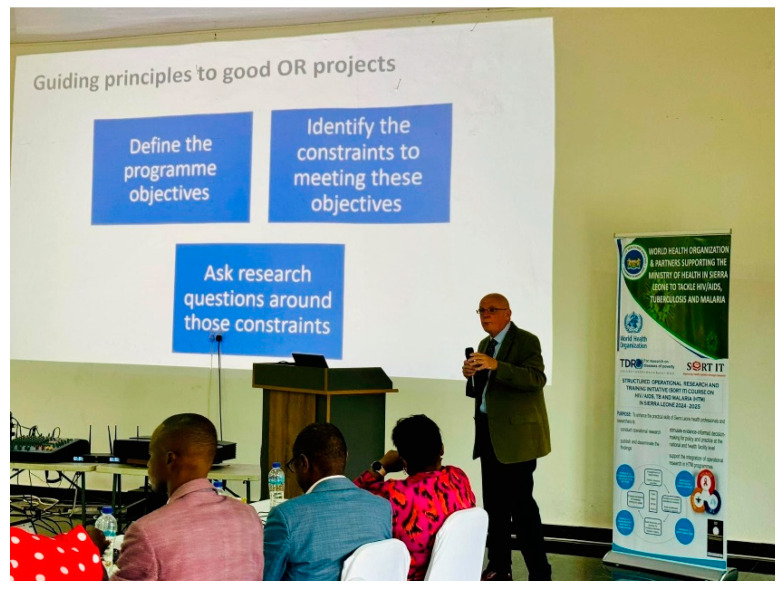
Guiding principles for good operational research questions (WHO/TDR).

Many fundamental gaps in knowledge are discernible in Table 1, Table 2, Table 3 and Table 4, and programmatic constraints are likely to underly the lack of progress (or lack of data) identified in Table 4. These gaps and constraints can be addressed using operational research. The Structured Operational Research and Training Initiative (SORT IT), a global partnership-based initiative led by the WHO/TDR, aims to support countries in conducting operational research (OR) on their own public health priorities, build a sustainable OR capacity in their public health programs and utilize the results of OR to improve public health program performance. The guiding principles in formulating relevant OR projects, as promoted by SORT IT, are as follows: define the program objectives; identify the constraints to meeting these objectives; and ask research questions around these constraints (see Figure 1).

## 5. This Special Issue on Tropical Medicine and Infectious Diseases Exemplifies How OR Can Be Used to Address Knowledge Gaps and Programmatic Constraints in NTD Control in West Africa

This Special Issue collates nine reports on operational NTD research supported by the WHO/TDR and conducted by public health program staff in Burkina Faso, Guinea and Senegal [34]. SORT IT’s guiding principles in formulating OR projects were instrumental in ensuring that the research conducted was of value to the NTD programs involved. Six of the studies were conducted in Guinea, two in Burkina Faso and one in Senegal. Two of the included manuscripts address snakebite envenomation, while two address leprosy. One study each focuses on lymphatic filariasis, onchocerciasis and trachoma. The study conducted in Senegal focuses on skin NTDs as a group, and another, conducted in Guinea, addresses issues related to preventive chemotherapy for NTDs in general. The submission and/or publication of the collected papers was followed by SORT IT training on knowledge management and research communication, providing the researchers with tools (including evidence briefs) with which they could inform a variety of stakeholders about their research and advocate for policy and/or practice change if appropriate.

## 6. Going Forward

**Figure 2:**
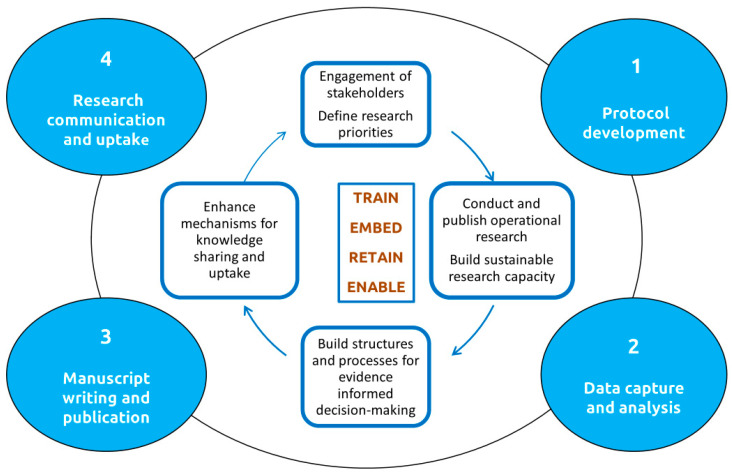
The SORT IT cycle.

We urge all national NTD control/elimination programs to consider how operational research could be utilized to enhance their program’s performance and help them achieve the 2030 goals of the NTD roadmap. SORT IT offers a unique approach to identify operational constraints or gaps in knowledge, such as those detailed in Table 1, Table 2, Table 3 and Table 4, and to formulate pertinent, policy/practice-relevant research questions. The approach enables even those who have never been involved in such studies before to conduct OR around research questions, publish their findings in reputable, peer-reviewed, open access journals and effectively communicate their research findings and their implications to a wide variety of stakeholders (Figure 2) [35]. For more information on SORT IT, please visit the SORT IT operational research and training website [35].

## Figures and Tables

**Table 1:** NTDs (in alphabetical order: B–H), WHO roadmap goals, and the current status of each NTD in each West African country.

	NTD	Goal by 2030	Benin	Burkina Faso	Cape Verde	Cote d’Ivoire	The Gambia	Ghana	Guinea	Guinea-Bissau	Liberia	Mali	Mauritania	Niger	Nigeria	Senegal	Sierra Leone	Togo
1	Buruli Ulcer [3]	Control	CE	PU	NE	CE	NE	CE	PU	NE	CE	NE	NE	NE	CE	PU	PU	CE
2	Chagas Disease [4,5]	Elimination as a public health problem	No vectorial transmission but an increasing possibility of congenital and blood transfusion-related transmission as a result of human migration. No data.															
3	Chikungunya and Dengue [6,7]	Control (serotypes)	CP CD (ND)	– CD (1, 2, 3)	– CD (1, 3)	CP CD (1, 3)	– –	– CD (ND)	CP CD (ND)	– –	– SD	– CD (1, 3)	– CD (ND)	– SD	CP ND	CP CD (ND)	CP –	– –
4	Dracunculiasis [8,9]	Eradication No. of cases reported (2023)	CF 2009	CF 2011	0	CF 2013	0	CF 2015	0	0	0	1	CF 2009	CF 2013	CF 2013	CF 2004	0	CF 2011
5	Echinococcosis [10]	Control	En	En	SE	En	ND	En	SE	SE	SE	En	En	En	SE	SE	SE	SE
6	Foodborne Trematodiases [11]	Control	P	P	ND	FP	ND	ND	P	ND	P	F	ND	F	FP	ND	P	ND
7	Human African Trypanosomiasis (HAT) [9,12,13]	HAT gambiense—the elimination/interruption of transmission No. of new cases reported (2023)	VEP 2022	0	NE	VEP 2020	0	VEP 2023	24	ND	0	0	0	0	0	0	0	VEP 2020
		HAT rhodesiense—elimination as a public health problem	NE	NE	NE	NE	NE	NE	NE	NE	NE	NE	NE	NE	NE	NE	NE	NE

**Legend for Table 1 (in alphabetical order). CD**: confirmed cases of dengue (with serotypes present). **CE**: currently endemic. **CF**: certified free-of-disease transmission. **CP**: chikungunya virus is known to be present. **En**: endemic. **F**: endemic for fascioliasis. **FP**: endemic for fascioliasis and paragonimiasis. **ND**: no WHO data. **NE**: non-endemic. **P**: endemic for paragonimiasis. **PU**: previously endemic, with current status unknown. **SD**: suspected cases of dengue. **SE**: suspected to be endemic. **VEP**: validated as eliminated as a public health problem, with the year.

**Table 2:** NTDs (in alphabetical order: L–R), WHO roadmap goals, and the current status of each NTD in each West African country.

	NTD	Goal by 2030	Benin	Burkina Faso	Cape Verde	Cote d’Ivoire	The Gambia	Ghana	Guinea	Guinea-Bissau	Liberia	Mali	Mauritania	Niger	Nigeria	Senegal	Sierra Leone	Togo
8	Leishmaniasis [14,15]	Visceral—elimination as a public health problem Cutaneous—control	Na Na	Na En	Na Na	En En	PC PC	Na En	Na En	Na En	Na Na	Na En	En En	En En	PC En	En En	Na Na	Na Na
9	Leprosy [16]	The elimination/interruption of transmission No. of new cases in individuals <15 years of age (2023)	7	9	3	42	0	7	6	0	11	1	0	38	151	34	10	3
10	Lymphatic Filariasis [9,17]	Elimination as a public health problem	US	MD	NE	MD	NE	MD	MD	MD	MD	US	MD	MD	MD	MD	MD	VEP 2017
11	Mycetoma, Chromoblastomycoses and Other Deep Mycoses [18]	Control No. of mycetoma cases (according to the WHO for the latest year available (2019))	ND	ND	10–50	ND	ND	ND	ND	ND	ND	51–100	101–500	101–500	10–50	>500	ND	51–100
12	Noma [19]	Only added to the WHO’s list of NTDs in December 2023.	Latest WHO estimates (1998): 140,000 incident cases per year, mainly in children aged 2–6 years and in poor communities in sub-Saharan Africa. Case fatality rate: 90%. No WHO country data are available.															
13	Onchocerciasis [20,21]	The elimination/interruption of transmission	PR	PR	NE	PR	NE	PR	PR	PR	PR	PR	NE	NPR	PR	NPR	PR	PR
14	Rabies [22]	Elimination as a public health problem	HR	HR	ND	HR	ND	HR	HR	HR	HR	HR	HR	HR	HR	HR	HR	HR

**Legend for Table 2 (in alphabetical order). En**: endemic. **HR**: dog-mediated human rabies is present. **MD**: mass drug administration is ongoing as of 2024. **Na**: no autochthonous cases reported (2023). **ND**: no WHO data. **NE**: non-endemic. **NPR**: no preventive chemotherapy required. **PC**: previously reported cases. **PR**: preventive chemotherapy required. **US**: under surveillance. **VEP**: validated as eliminated as a public health problem, with the year.

**Table 3:** NTDs (in alphabetical order: S–Y), WHO roadmap goals, and the current status of each NTD in each West African country.

	NTD	Goal by 2030	Benin	Burkina Faso	Cape Verde	Cote d’Ivoire	The Gambia	Ghana	Guinea	Guinea-Bissau	Liberia	Mali	Mauritania	Niger	Nigeria	Senegal	Sierra Leone	Togo
15	Scabies and Other Ectoparasitoses [23]	Control	ND	ND	ND	ND	ND	ND	ND	ND	ND	ND	ND	ND	ND	ND	ND	ND
16	Schistosomiasis [24]	Elimination as a public health problem	PR	PR	NE	PR	PR	PR	PR	PR	PR	PR	PR	PR	PR	PR	PR	PR
17	Snakebite Envenoming [25,26]	Control No. of cases/average incidence per 100,000 people (deaths per year)	1682/ 14 (ND)	12,054/ 66 (242)	ND	ND	ND	11,080/ 45 (ND)	875/7 (49)	ND	ND	ND	ND	ND	ND	879/9 (ND)	670/8 (ND)	3001/44 (54)
18	Soil-Transmitted Helminthiases [27]	Elimination as a public health problem	PR	NPR	PR	PR	PR	NPR	PR	PR	PR	NPR	NPR	NPR	PR	PR	PR	PR
19	Taeniasis and Cysticercosis [28]	Control	En	En	En	En	SE	En	SE	En	SE	SE	NE	SE	En	En	SE	En
20	Trachoma [29]	Elimination as a public health problem	VEP 2023	KRI	TNI	KRI	VEP 2021	VEP 2018	KRI	KRI	TNI	VEP 2023	TNCE	KRI	KRI	KRI	TNI	VEP 2022
21	Yaws [30]	Eradication	CE	PU	NH	CE	PU	CE	PU	PU	CE	PU	NH	PU	PU	PU	PU	CE

**Legend for Table 3 (in alphabetical order). CE**: currently endemic. **En**: endemic. **KRI**: known to require interventions. **ND**: no WHO data. **NE**: non-endemic. **NH**: no previous history of the disease. **NPR**: no preventive chemotherapy required. **PR**: preventive chemotherapy required. **PU**: previously endemic, with current status unknown. **SE**: suspected to be endemic. **TNCE**: not thought to require interventions, with claims that it has been eliminated. **TNI**: not thought to require interventions. **VEP**: validated as eliminated as a public health problem, with the year.

**Table 4:** The progress of West African countries in attaining the goals of the WHO 2021–2030 NTD roadmap [9].

Progress toward 2030 Targets of WHO NTD Roadmap	Benin	Burkina Faso	Cape Verde	Cote d’Ivoire	The Gambia	Ghana	Guinea	Guinea-Bissau	Liberia	Mali	Mauritania	Niger	Nigeria	Senegal	Sierra Leone	Togo
**OVER-ARCHING INDICATORS FOR 2030 TARGET**																
No. of endemic NTDs eliminated to date	3	1	0	2	1	3	0	0	0	1	1	1	1	1	0	4
% reduction in people requiring interventions against NTDs (target: **90%**)	14.1	79.2	16.1	12.3	60.1	6.4	5.3	28.9	11.7	36.3	40.8	21.4	13.2	62.1	6.6	17.5
% reduction in DALYs * related to NTDs (target: **75%**)	21.5	15.8	−5.7	4.6	38.7	18.4	−51.6	10.5	0.6	13.0	12.7	−1.8	12.3	18.2	11.8	21.2
**CROSS-CUTTING INDICATORS FOR 2030 TARGET**																
**Integrated Approaches**																
% reduction in no. of deaths from vector-borne NTDs (target: **75%**)	ND	ND	ND	ND	ND	ND	ND	ND	ND	ND	ND	ND	ND	ND	ND	ND
Integrated treatment coverage index for preventive chemotherapy (target: **75%**)	75	82.0	59.0	58.0	1.0	63.0	62.0	45.0	72.0	86.0	3.0	56.0	46.0	20.0	85.0	87.0
**Multi-Sectoral Coordination**																
Access to at least basic water, sanitation and hygiene in areas endemic for NTDs (target: **100%**)	39.8	29.3	86.5	21.8	12.9	41.7	41.1	19.7	33.9	50.5	58.5	36.8	31.1	22.3	17.8	17.3
Proportion of NTDs integrated into national health strategies/plans (target: **75%**)	66.7	91.7	ND	ND	50.0	100	100	ND	87.5	100	ND	83.3	ND	100	66.7	88.9
**Universal Health Coverage**																
Proportion of NTDs for which interventions are included in packages of essential services and budgeted (target: **75%**)	ND	ND	ND	ND	0.0	0.0	0.0	ND	ND	100	ND	50.0	ND	33.3	33.3	22.2
Proportion of NTDs related to disability with guidelines for management within national health systems (target: **75%**)	25.0	88.9	ND	ND	33.3	100	100	ND	100	100	ND	60.0	ND	75.0	50.0	83.3
**Country Ownership**																
Proportion of relevant NTDs on which country reports (target: **75%**)	16.0	83.0	ND	ND	66.0	100	33.0	ND	100	100	ND	83.0	ND	100	100	66.0
Proportion of NTDs for which country collects and reports data disaggregated by gender (target: **75%**)	16.7	8.3	ND	ND	ND	100	22.2	ND	75.0	100	ND	16.7	ND	100	33.3	33.3

* DALYs: disability-adjusted life years. **ND**: no WHO data. Red font: data are not available or the 50% point has not been reached.
